# Intimal hyperplasia and adventitial angiogenesis originating from veins are vascular remodelling responses in large vessels after major lower extremity trauma induced ischemia

**DOI:** 10.1007/s10456-026-10089-x

**Published:** 2026-09-03

**Authors:** Adas Cepas, Paavo Saarela, Tuomas Komulainen, Tero Järvinen, Juha Kiiski, Ilkka Kaartinen

**Affiliations:** 1 Department of Musculoskeletal Surgery and Diseases, Faculty of Medicine and Health Technology, Tampere University Hospital and University of Tampere, Tampere, Finland; 2 Faculty of Medicine and Health Technology, Tampere University, Tampere, Finland

**Keywords:** Vascular remodelling, Neovascularization, Hypoxia, Angiogenesis, Large artery, Microsurgery

## Abstract

**Background:**

Total paucity exists on how large human blood vessels respond to ischemia. We explored the human vascular response to trauma-induced ischemia, with a particular focus on vascular wall remodelling.

**Methods:**

Arterial and venous wall samples were analysed from 40 patients undergoing soft tissue free flap reconstruction following lower extremity trauma. Patients were stratified into an early reconstruction group (median 6 days post-injury (IQR 4–8); n = 26) after high-energy lower extremity open fracture, and a late reconstruction group (median 54 days post-injury (IQR 31–155); n = 14) operated due to fracture non-union or fracture related infection. Injury-site arterial and venous biopsy samples were collected at the anastomosis site during reconstruction, while control samples were obtained from intact blood vessels at free flap donor sites. Quantitative histological and immunohistochemical analyses were performed using QuPath software.

**Results:**

The cohort consisted predominantly of working-age males (median age: early group 44 years, late 50 years; *p *= 0.72). Arterial intimal hyperplasia was significantly greater at injury sites than in the control arteries in both groups. Venous intimal thickening exceeded control levels by more than threefold. Adventitial angiogenesis was primarily observed in veins in the early reconstruction group, and it was more pronounced in both arteries and veins of the late group compared to the early group.

**Conclusions:**

Our findings demonstrate that major lower extremity trauma and trauma-induced ischemia trigger active remodelling across all layers of the vascular wall. The most prominent structural changes are linked to rapid onset of intimal hyperplasia and adventitial angiogenesis originating from the venous system.

**Supplementary Information:**

The online version contains supplementary material available at https://doi.org/10.1007/s10456-026-10089-x.

## Introduction

Oxygen is fundamental for most living organisms, and the delivery of oxygen is a physiological challenge for humans. Accordingly, oxygen deprivation, hypoxia, is a critical component of many human diseases. The knowledge on vascular response and remodelling following major ischemic insult to the surrounding tissues is very limited and lacks fundamental background to date. The reconstructive surgeons have noticed early onset of macroscopic vascular and perivascular changes that start within the first days after ischemic insult to the extremities. Progressive perivascular “fibrosis” with loss of normal easy planes of vascular dissection and vessel wall fragility are the macroscopical changes taking place in blood vessels reacting to hypoxia. Moreover, it is thought that vessels affected by trauma are more prone to severe vasospasm and lack thromboresistant properties [1–4]. While ischemia likely represents an important driver of vascular changes, post-traumatic vascular remodelling is probably multifactorial, involving direct mechanical injury, inflammatory responses, altered hemodynamic forces, and tissue repair mechanisms. However, beyond macroscopic changes, how human large vessels respond to severe traumatic injury at a more fundamental level remains largely unknown. Accordingly, the current understanding of vascular remodelling following ischemic injury is based on experimental animal models and cadaveric studies. These studies have primarily evaluated vascular wall changes induced by endovascular and open vascular procedures. Iatrogenic vascular injury to blood vessels leads to intimal hyperplasia and extracellular matrix (ECM) formation within intima, resulting in lumen stenosis. [5–10] However, vascular changes induced in blood vessels by interventional or experimental surgical injury may not accurately reflect those caused by trauma-induced hypoxia. To date, there is a complete lack of studies analysing early and late changes in human blood vessels following major ischemic insult.

To address this knowledge gap, we have performed a prospective trial in which arterial and venous biopsies were collected from the injury site of high-energy lower extremity open fractures at the time of fracture repair and free tissue transfer. These samples were compared to uninjured control samples obtained from arteries and veins from the free flap donor sites of the same individuals. The variable interval from injury to reconstruction and sample collection provided us with unique sets of samples, enabling investigation of immediate vascular response to trauma as well as subsequent dynamics of trauma-induced ischemia.

## Materials and methods

### Patients and surgical procedures in the prospective trial

This prospective comparative study investigating human vessel walls was conducted at Tampere University Hospital and Tampere University, Finland. The protocol for this study was approved by the Institutional Ethics Review Board (No. R20024). A total of 40 patients underwent soft tissue free flap reconstruction for the lower extremity. To evaluate vascular remodelling during the early and late phases following injury, patients were stratified into two groups: early reconstruction (ER) and late reconstruction (LR). The ER group consisted of 26 patients operated due to acute high-grade open fracture of the lower extremity. The median interval from injury to free flap reconstruction was 6 days (IQR 4–8; range 1–13 days). These patients received initial and definitive fracture management including free flap transfer according to institutional orthoplastic treatment algorithm. [11] In contrast, the LR group included 14 patients following lower extremity fracture who presented with complications following failed primary fracture treatment, and who required free-flap reconstruction for wound complication or for compromised soft-tissues. The median interval from injury to free flap reconstruction in this group was 54 days (IQR 31–155; range 17–6570 days). All but one patient in this group were referred to our center from outside hospitals. None had undergone free-flap surgery prior to enrolment.

**Fig. 1:**
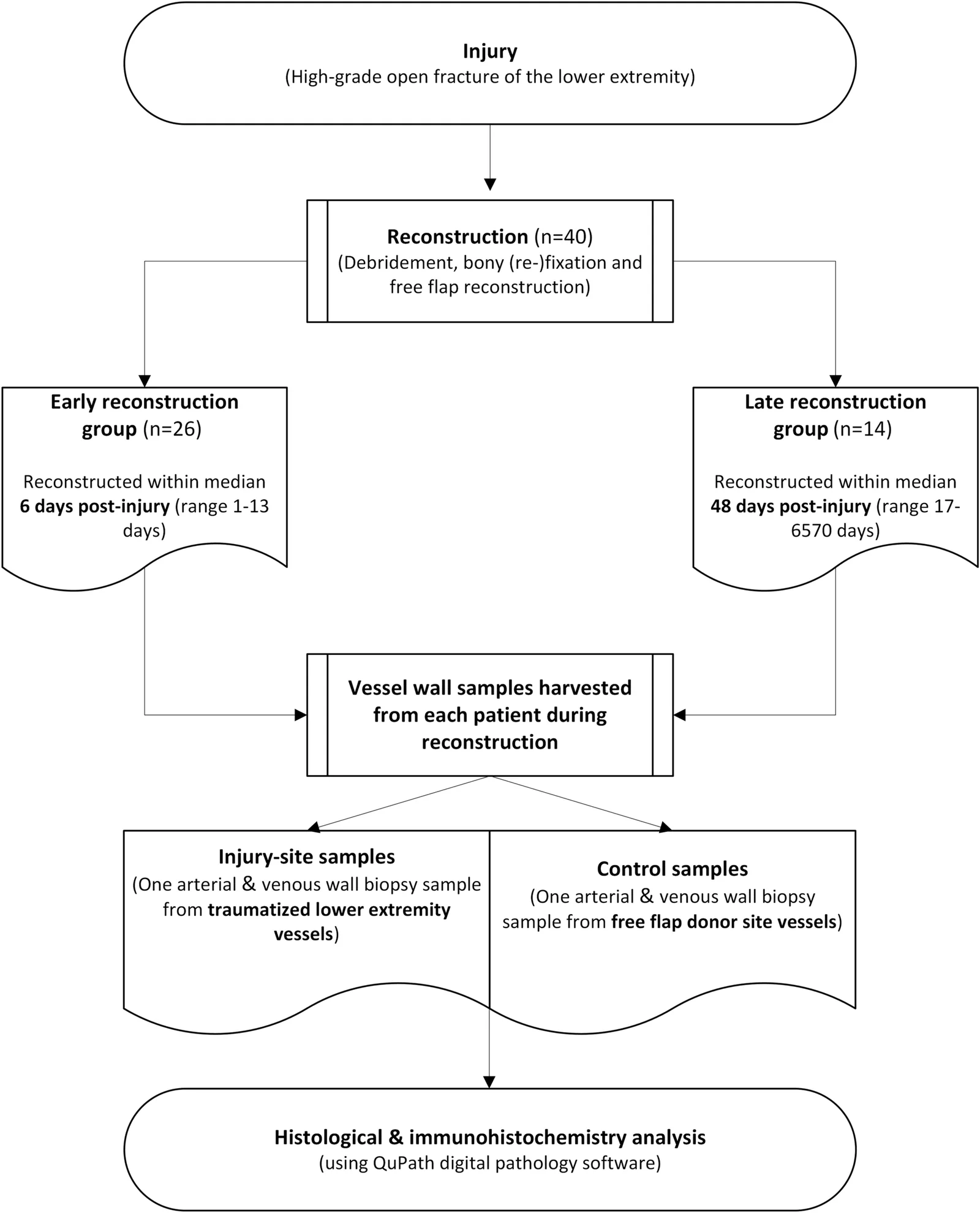
Flowchart describing study cohort, grouping and protocol

During free flap reconstruction one arterial and one venous wall sample were harvested from the injury site and size-matched uninjured control samples (one arterial and one venous wall sample) were harvested from the free flap donor site of the same patient. Injury-site vessel wall samples were harvested at the anastomotic site where vessels were deemed suitable for microvascular anastomosis in proximity to the fracture (Fig. 1).

**Fig. 2:**
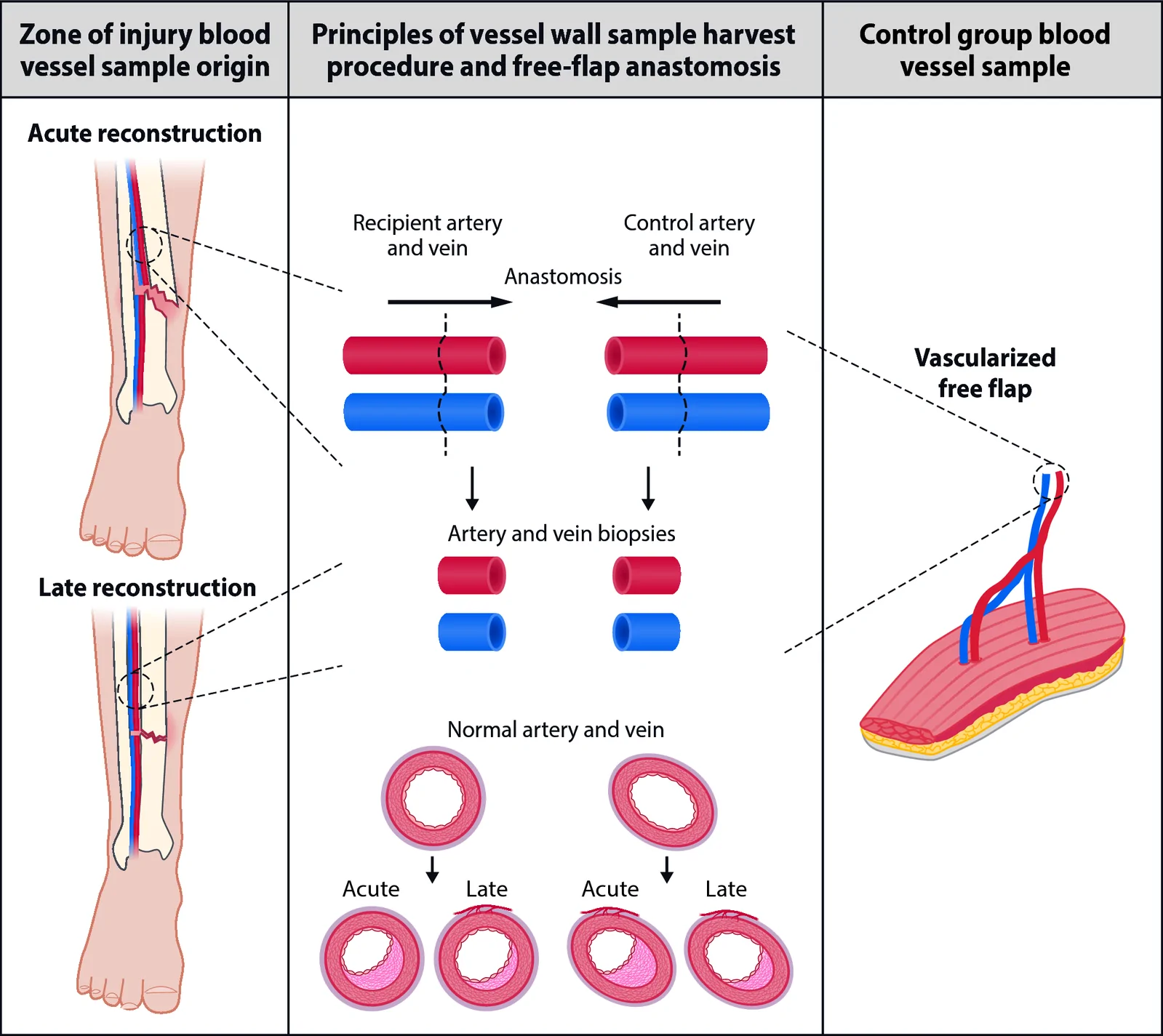
Vessel wall sample origin, harvest for the analysis and vascular anastomoses

Free-flap arterial anastomoses were performed either end-to-end or end-to-side based on clinical judgement and vessel patency. Venous anastomoses were always performed end-to-end using venous coupler (Fig. 2).

Singular unplanned readmissions occurred in one patient per group. In the early reconstruction group one patient underwent below-knee amputation due to deep infection 45 days after reconstruction. In the late reconstruction group one patient was readmitted a year after failed attempt to eradicate osteomyelitis and required debridement and secondary reconstruction. However, these cases enabled additional vessel wall sampling at later time points providing valuable samples harvested from the same region in different timeframes.

### Histology and morphometric analysis

Vessel wall samples were harvested as circumferential cross-sections and fixed in 10% formalin at room temperature for 24 h and further processed to paraffin blocks. 5-µm thick histological sections from the harvested vessels were prepared and stained with haematoxylin and eosin, Elastic Stain Kit (Verhoeff Van Gieson, Abcam, Cambridge, UK), and Masson’s trichrome (Sigma, Merck Life Science, Espoo, Finland). The histological sections were scanned with NanoZoomer S360 (Hamamatsu Photonics, Hamamatsu City, Japan) or Olympus VS200 Slide Scanner Microscope (Olympus, Tokyo, Japan). Microscopy and the open-source digital pathology software QuPath (RRID: SCR_018257) were used for pathology image analysis. [12] The intima was identified as the layer between the endothelial surface and the internal elastic lamina. The media was defined as the circumferential smooth muscle cell layer located between the internal and external elastic lamina. The adventitia was identified as the outer connective tissue layer containing collagen fibers, fibroblasts, and, when present, vasa vasorum and peripheral nerves.

Intimal and medial thicknesses were measured on Elastic Van Gieson-stained circumferential cross-sections at the site of maximal vessel wall thickening using *“line”* feature in digital pathology software (QuPath). Intimal thickness was measured as the perpendicular distance from the luminal surface to the internal elastic lamina, whereas medial thickness was measured as the perpendicular distance from the internal elastic lamina to the external elastic lamina.

The extracellular matrix (ECM) fraction in the intimal and medial layers was quantified using the semi-automated, artificial intelligence-guided pixel classifier function in QuPath. The classifier was trained on multiple images before its application for the analysis. To detect ECM and smooth muscle cells, specific thresholds were selected manually by one researcher (PS) for blue (collagen) and red-stained (smooth muscle cells) components in the Masson’s trichrome-stained sections. The ECM fraction of the vessel walls was calculated as a percentage of the annotated area. While separate intimal and medial annotations were defined for arteries, a single combined annotation was used for veins.

### Immunohistochemistry (IHC)

IHC staining was performed on 6-µm-thick paraffin sections using standard IHC protocols. [13–16] The following primary antibodies were used: mouse anti-human CD31 (cluster of differentiation 31, DakoCytomation, Glostrup, Denmark, Cat# M0823, RRID:AB_2114471), rat anti-Ki67 (TEC-3; Agilent Cat# M7249, RRID:AB_2250503) at 1:200, and rabbit anti-α-smooth muscle actin (α-SMA) (Abcam, Cambridge, UK, Cat# ab5694, RRID:AB_2223021) at 1:100, followed by the appropriate horseradish peroxidase-conjugated secondary antibodies (Nichirei Biosciences Inc., N-Histofine, universal immuno-peroxidase polymer, anti-mouse and -rabbit immunohistochemical staining reagent, Tokyo, Japan). [14–16] The blocking reagents used for IHC were S2O23 REAL and S0809 Antibody Diluent (DakoCytomation). The peroxidase-reactive chromogen used was diaminobenzidine (K3465, DAKO, Agilent Technologies).

### Virtual microscopy and quantitative analyses

All the IHC stained sections were scanned as digital images using an Olympus VS200 Slide Scanner Microscope (Olympus, Tokyo, Japan). Quantitative analyses were performed with QuPath version 0.5.1, open-source pathology image analysis software (RRID: SCR_018257). [12] For each blood vessel biopsy sample, the intimal, medial and adventitial layers were manually identified and annotated as separate regions of interest (ROIs) by a single researcher (PS). The same ROIs were applied consistently across all IHC staining for each specimen. Depending on the antibody used, the fraction of positively stained cells among the detected cells, the density of positively stained cells, or the area of positive staining (positively stained pixels) was quantified using QuPath’s automated analysis tools. Immunohistochemical results were expressed as the number of positive cells or micro vessels per mm^2^ of the corresponding annotated ROI or as the percentage of positively stained area, as appropriate. The threshold for positivity was selected manually by a single researcher (PS), and the same threshold was used across all the samples in each antibody group. Not every biopsy sample contained all the layers of the blood vessel wall. All the IHC, scanning and quantification processes were performed in a blind fashion. All the samples were coded during our study, and the code was decrypted only after the analyses.

### Immunofluorescence

Double-immunofluorescence staining was performed on 5 µm thick paraffin sections. Different antigen retrieval methods were tested for each antibody to identify the ideal method. The antigen retrieval was performed in 10 mM TRIS–HCl- 1 mM EDTA and 0.05% Tween 20 (pH 9) using a pressure cooker (120 °C). The following primary antibodies were used: 1:100 rabbit anti-α-smooth muscle actin (α-SMA) (Abcam, Cambridge, UK, Cat# ab5694, RRID: AB_2223021) and 1:100 mouse anti-human CD31 (cluster of differentiation 31, DakoCytomation, Glostrup, Denmark, Cat# M0823, RRID: AB_2114471). BSA in PBS was used as a blocking agent followed by 3% hydrogen peroxide treatment of the tissue sections. The secondary antibodies used were 1:200 Alexa 594 goat anti-rabbit (Invitrogen Carlsbad, CA, USA, Cat# 111–587-003, RRID: AB_2338071) and Histofine anti-mouse (Nichirei Biosciences Inc., Tokyo, Japan, Cat# 414132F) followed by AlexaFluor 488 Tyramide SuperBoost according to the manufacturer´s instructions (B40943, Invitrogen, Carlsbad, CA, USA). The staining procedure was completed with a stop solution and 1:500 DAPI (MBD0015, Sigma-Aldrich, St. Louis, MO, USA). The samples were mounted using Fluoroshield™ with 1,4-Diazabicyclo [2.2.2]octane mounting medium (Sigma-Aldrich, St. Louis, MO, USA). All the immunofluorescence-stained sections were scanned to digital images via an Olympus VS200 Slide Scanner Microscope (Olympus, Tokyo, Japan).

### Statistical analysis

All statistical analyses were performed with IBM SPSS Statistics version 24.0 (IBM Corp., Armonk, NY, USA). Based on visual assessment of data distribution, cell proliferation was considered to follow a lognormal distribution. Consequently, zero values were replaced with one-half of the smallest nonzero value detected in the entire dataset prior to base−10 logarithmic transformation. Cell proliferation data are presented as geometric means with 95% confidence intervals (CI). All other continuous variables were treated as non-parametric and are presented as medians and interquartile ranges (IQR). Comparisons between injury-site and control samples obtained from the same patient at the same time point were analysed using Wilcoxon signed-rank test for paired observations. Comparisons between the early reconstruction and late reconstruction groups were analysed using Mann–Whitney U test, as these groups represented independent samples. Categorical variables were compared using Pearson chi-square test. All statistical tests were two-sided and a *p*-value of 0.05 was considered statistically significant.

## Results

### Study population

The study cohort consisted of predominantly working-age males, with median age of 44 years (IQR 29–57) in early and 50 years (IQR 30–59) in late reconstruction groups, respectively (*p *= 0.72). The prevalence of arterial hypertension was 4% among early and 7% among late reconstruction group patients (*p *= 0.71). 19% early and 28% late reconstruction group patients were active smokers (*p *= 0.35). No free flap losses, late amputations or major vascular complications were recorded in our cohort (Table S1. Supplemental digital content).

### Histological examination of arterial and venous walls

**Fig. 3:**
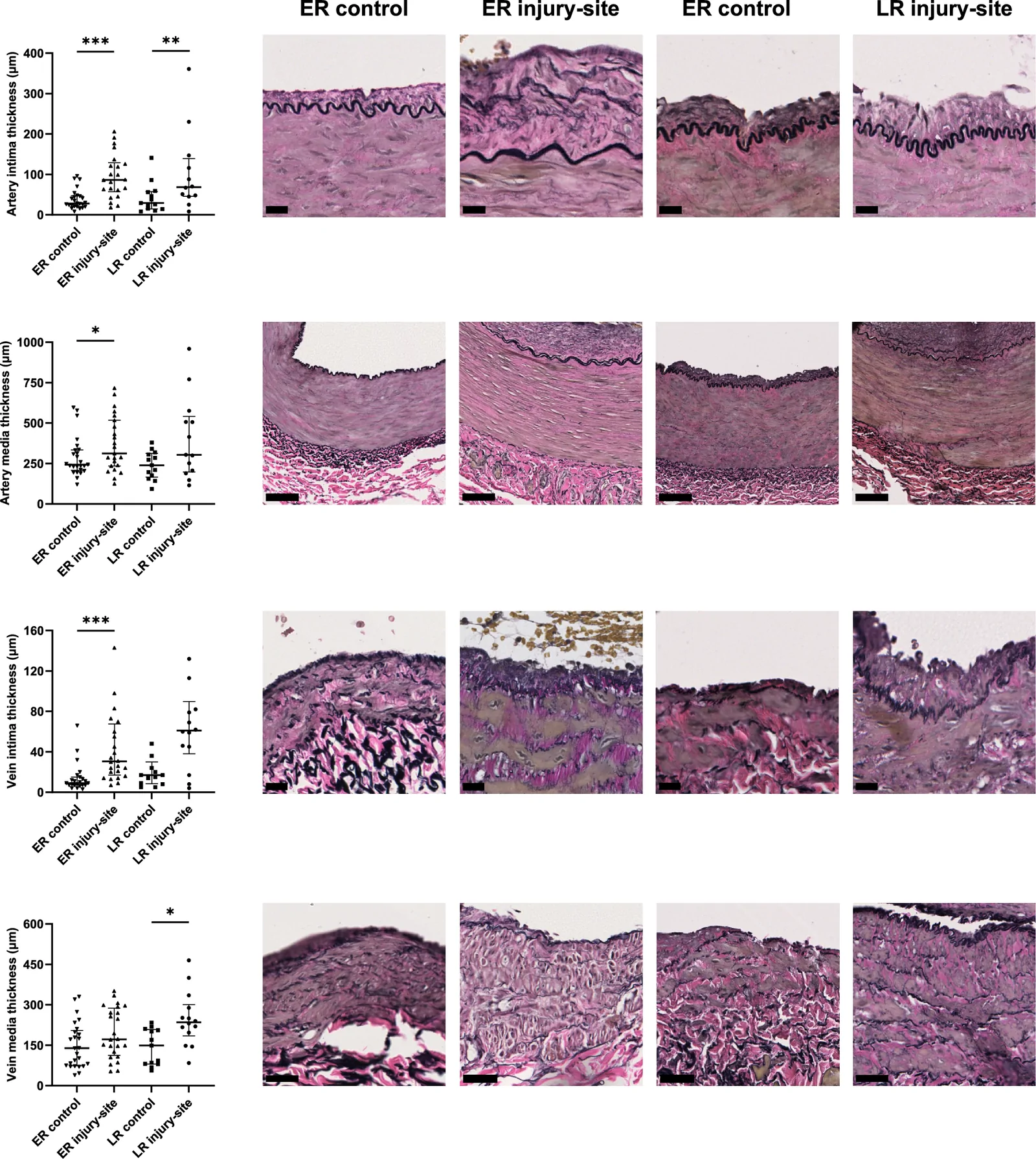
Morphometric analysis of intimal and medial thickness of vessel walls in early (ER) and late reconstruction (LR) groups and their controls. Histological sections were stained with Verhoeff Van Gieson stain and intimal and medial thicknesses were defined by measuring each layer at the thickest part. Representative images of all groups are provided adjacent to each graph. Scale bars: 100 μm for arterial media, 50 μm for venous media and 20 μm for arterial and venous intima. ^∗^*P* < 0.05, ^∗∗^*P* < 0.01 and ^***^P < 0.001. Middle line=median and whiskers=interquartile range (IQR). Statistical tests are reported in Tables 1 and 4. Images were taken with Hamamatsu NanoZoomer S360

**Fig. 4:**
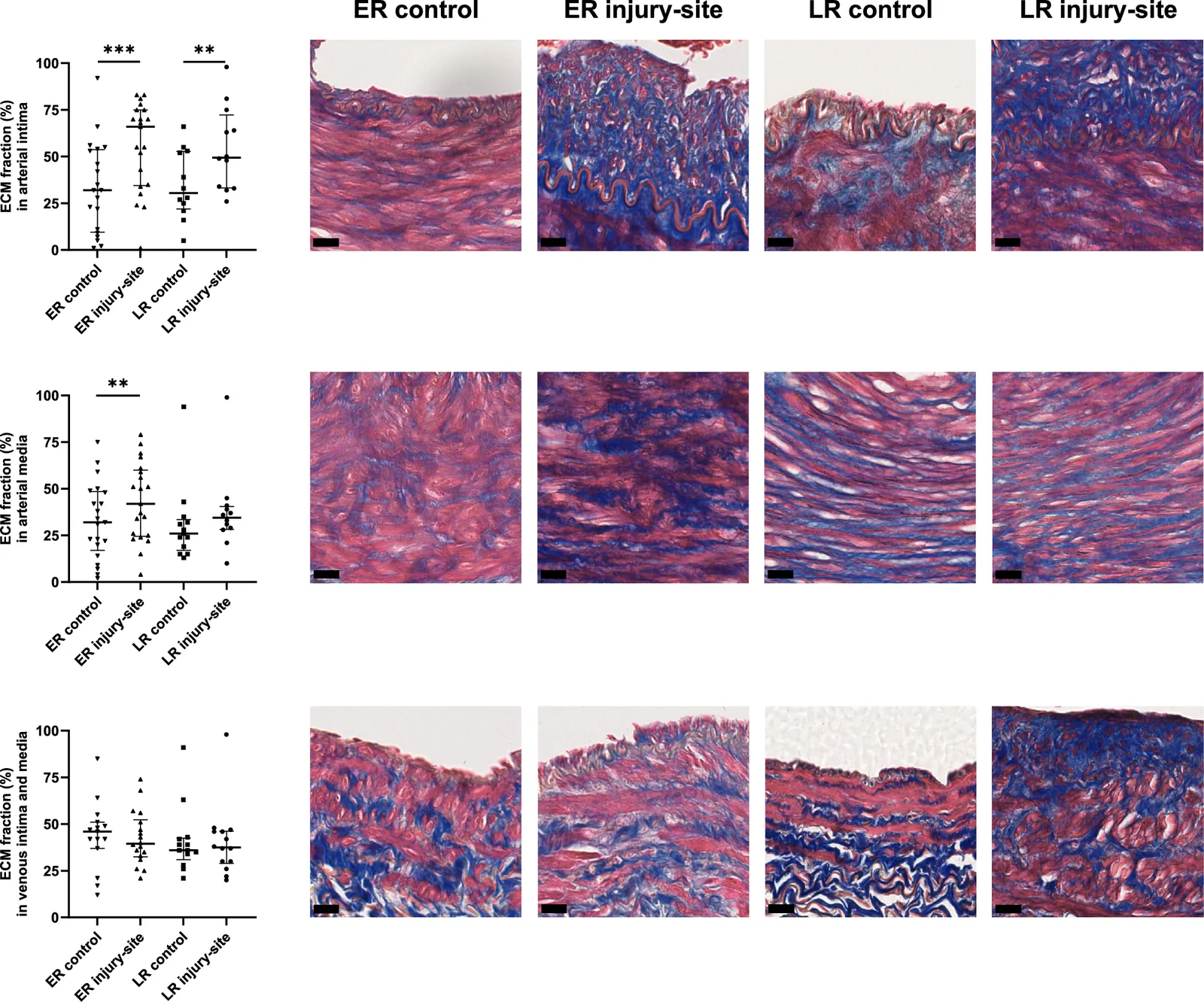
Extracellular matrix (ECM) formation in arterial and venous walls in early (ER) and late reconstruction (LR) groups and their controls. The amount of ECM (blue) was defined from Masson’s trichrome stain and is given as a fraction of annotated area. ECM fraction was calculated in arterial intima and media separately. In veins, intima and media were combined for the analysis. Representative images of Masson’s trichrome stained sections are provided adjacent to each graph. Scale bars: 20 μm. ^*^P < 0.05, ^**^P < 0.01 and ^***^P < 0.001. Middle line=median and whiskers=interquartile range (IQR). Statistical tests are reported in Tables 2 and 3. Images were taken with Hamamatsu NanoZoomer S360

Morphometric analysis of arterial walls revealed that the intima in injury-site samples was thicker than in the controls (87 µm vs. 28 µm, *p* < 0.01 in early reconstruction group and 69 µm vs. 29 µm, *p *< 0.01 in late reconstruction group) (Fig. 3; Table 1). Moreover, the amount of ECM in arterial intima was twofold higher in the injury-site samples than in the control samples in the early reconstruction group (66% vs. 32%, *p *< 0.01). This was also evident in late reconstruction group samples (50% vs. 31% *p *< 0.01, respectively) (Fig. 4). Venous intima was more than threefold thicker in injury-site samples than in the controls in both study groups, however, no statistically significant differences were observed in ECM area in veins (Tables 1 and 2). The comparative analysis of injury-site and control samples between early and late reconstruction groups did not reveal any significant differences in arteries or veins (Tables 3 and 4).

**Table 1:** Comparison of morphometric characteristics between control and injury-site samples in the early reconstruction (ER) and late reconstruction (LR) groups

	ER control	ER injury-site	*p*-value	LR control	LR injury-site	*p*-value
Artery intima thickness	28 (21–47)	87 (57–129)	< 0.01**	29 (15–58)	69 (46–140)	< 0.01**
Artery media thickness	243 (208–334)	312 (235–518)	0.03*	239 (166–312)	304 (198–542)	0.06
Artery intima/media ratio	0.12 (0.1–0.2)	0.24 (0.12–0.4)	< 0.01**	0.16 (0.1–0.18)	0.18 (0.13–0.3)	0.20
Vein intima thickness	9 (7–15)	31 (17–68)	< 0.01**	17 (9–30)	61 (38–90)	0.07
Vein media thickness	139 (74–205)	172 (113–288)	0.28	149 (81–209)	236 (185–301)	0.04*
Vein intima/media ratio	0.07 (0.04–0.15)	0.22 (0.12–0.29)	< 0.01**	0.15 (0.10–0.25)	0.24 (0.15–0.53)	0.83

Thickness µm

Data are presented as median (IQR). Statistical comparisons were performed using the Wilcoxon signed rank test. Statistical significance was defined as *P *< 0.05 (*) and *P *< 0.01 (**)

**Table 2:** Comparison of extracellular matrix formation between control and injury-site samples in the early reconstruction (ER) and late reconstruction (LR) groups

	ER control	ER injury-site	*p*-value	LR control	LR injury-site	*p*-value
Artery intima	32 (10–54)	66 (35–75)	< 0.01**	31 (22–53)	50 (33–72)	< 0.01**
Artery media	32 (17–49)	42 (25–60)	< 0.01**	26 (17–34)	35 (28–41)	0.14
Vein intima and media	46 (37–51)	40 (33–52)	0.47	36 (31–43)	38 (29–46)	0.80

Percentage %

Data is presented as median (IQR). Statistical comparisons were performed using the Wilcoxon signed rank test. Statistical significance was defined as *P *< 0.05 (*) and *P *< 0.01 (**)

**Table 3:** Comparison of extracellular matrix formation in vessel walls between the early reconstruction (ER) and late reconstruction (LR) groups

	ER injury-site	LR injury-site	*p*-value	ER control	LR control	*p*-value
Artery intima	66 (35–75)	50 (33–72)	0.78	32 (10–54)	31 (22–53)	0.84
Artery media	42 (25–60)	35 (28–41)	0.14	32 (17–49)	26 (17–34)	0.64
Vein intima and media	40 (33–52)	38 (29–46)	0.52	46 (37–51)	36 (31–43)	0.20

Percentage %

Data are presented as median (IQR). Statistical comparisons were performed using the Mann–Whitney U test. Statistical significance was defined as *P *< 0.05 (*) and *P *< 0.01 (**)

**Table 4:** Comparison of morphometric characteristics of vessel walls between the early reconstruction (ER) and late reconstruction (LR) groups

	ER injury-site	LR injury-site	*p*-value	ER control	LR control	*p*-value
Artery intima thickness	87 (57–129)	69 (46–140)	0.74	28 (21–47)	29 (15–58)	0.86
Artery media thickness	312 (235–518)	304 (198–542)	0.97	243 (208–334)	239 (166–312)	0.36
Artery intima/media ratio	0.24 (0.12–0.4)	0.18 (0.13–0.3)	0.64	0.12 (0.1–0.2)	0.16 (0.1–0.18)	0.28
Vein intima thickness	31 (17–68)	61 (38–90)	0.15	9 (7–15)	17 (9–30)	0.08
Vein media thickness	172 (113–288)	236 (185–301)	0.23	139 (74–205)	149 (81–209)	0.99
Vein intima/media ratio	0.22 (0.12–0.29)	0.24 (0.15–0.53)	0.64	0.07 (0.04–0.15)	0.15 (0.10–0.25)	0.08

Thickness µm

Data are presented as median (IQR). Statistical comparisons were performed using the Mann–Whitney U test. Statistical significance was defined as *P *< 0.05 (*) and *P *< 0.01 (**)

### Proliferative activity of ischemic vascular walls

**Fig. 5:**
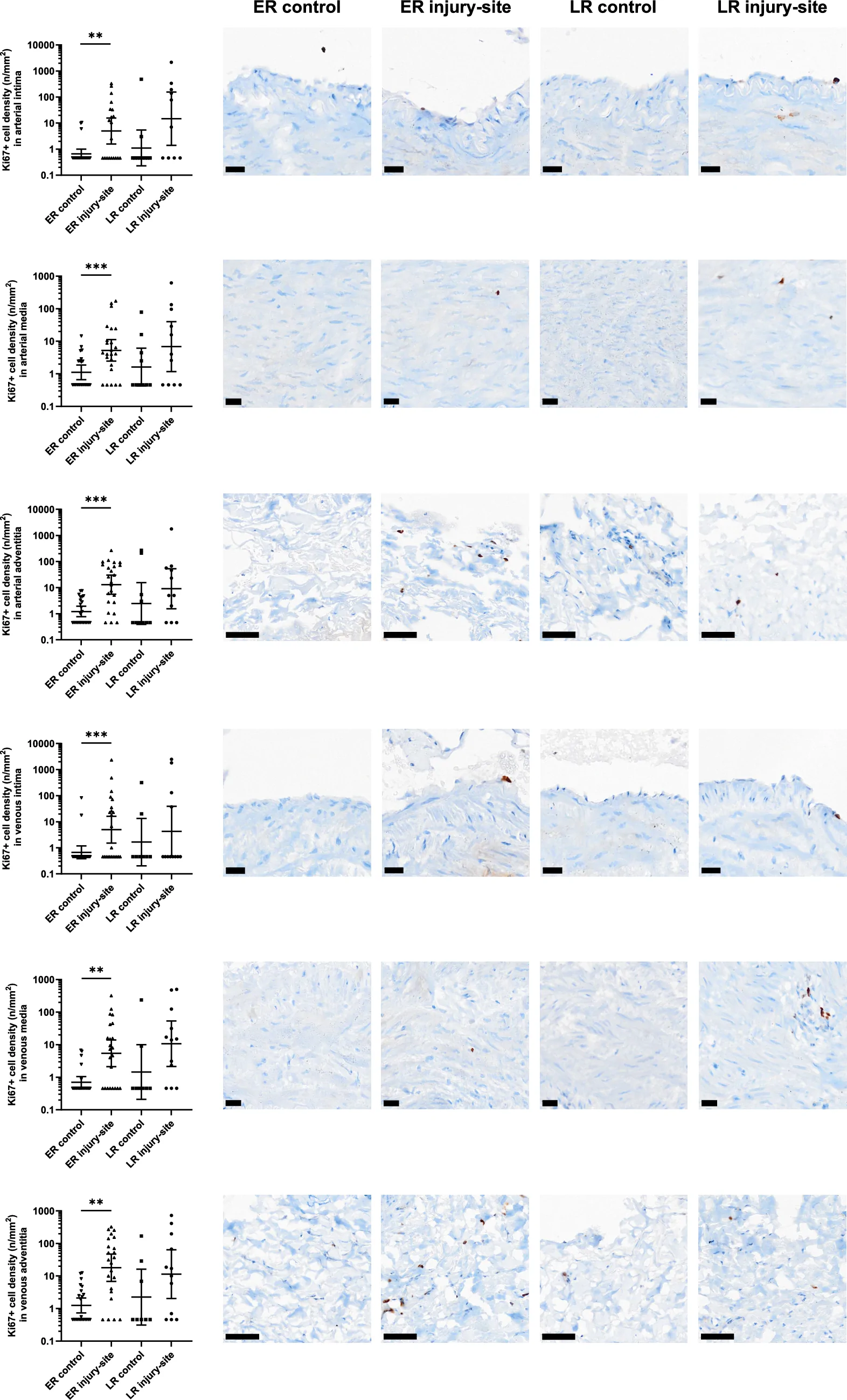
Proliferative activity in arterial and venous walls of early (ER) and late reconstruction (LR) groups and their controls. Proliferating cells (Ki67 +) were identified with a Ki67-antibody and quantified in each layer of the vessels. Representative images of all groups are provided adjacent to each graph. Scale bar: 50 μm for arterial and venous adventitia and 20 μm for arterial and venous media and intima. ^*^P < 0.05, ^**^P < 0.01 and ^***^P < 0.001. Middle line=geometric mean and whiskers=95% confidence interval (CI) of geometric mean. Statistical tests are reported in Tables 5 and 6. Images were taken with Olympus VS200 Slide Scanner Microscope

The proliferative activity in vascular wall cells was surprisingly low following major soft tissue injury (Fig. 5). However, all vessel wall layers showed higher Ki67 activity in early reconstruction group, especially in intimal and adventitial layers of both arteries and veins, than in their control samples. Proliferative activity was noticeably higher only in venous adventitial layer of the injury-site vessels than in the controls in late reconstruction group (Table 5). Similarly to morphometric characteristics, comparative analysis of injury-site and control samples did not show any statistically significant differences in cell proliferative activity in arteries and veins between the study groups (Table 6).

**Table 5:** Comparison of KI-67-positive cell proliferation between control and injury-site samples in the early reconstruction (ER) and late reconstruction (LR) groups

	ER control	ER injury-site	*p*-value	LR control	LR injury-site	*p*-value
Artery intima	0.66 (0.43–1)	4.93 (1.57–15.57)	< 0.01**	1.11 (0.23–5.40)	14.63 (1.38–154.62)	0.13
Artery media	1.11 (0.66–1.86)	5.25 (2.45–11.27)	< 0.01**	1.62 (0.43–6.12)	6.87 (1.17–40.31)	0.06
Artery adventitia	1.22 (0.76–1.94)	13.08 (5.62–30.42)	< 0.01**	2.46 (0.39–15.49)	9.06 (1.56–52.55)	0.15
Vein intima	0.67 (0.38–1.18)	4.99 (1.531–16.24)	< 0.01**	1.66 (0.21–13.42)	4.31 (0.47–39.36)	0.75
Vein media	0.70 (0.47–1.05)	5.43 (2.12–13.91)	< 0.01**	1.45 (0.21–9.96)	10.71 (2.14–53.47)	0.22
Vein adventitia	1.24 (0.73–2.12)	17.94 (6.84–47.06)	< 0.01**	2.26 (0.31–16.33)	11.42 (2.04–64.15)	0.06

Ki67+ cells/mm^2

Data are presented as geometric means with 95% confidence intervals (95% CI). Statistical comparisons were performed using the Wilcoxon signed rank test. Statistical significance was defined as *P *< 0.05 (*) and *P *< 0.01 (**)

**Table 6:** Comparison of Ki-67-positive cell proliferation in vessel walls between the early reconstruction (ER) and late reconstruction (LR) groups

	ER injury-site	LR injury-site	*p*-value	ER control	LR control	*p*-value
Artery intima	4.93 (1.57–15.57)	14.63 (1.38–154.62)	0.36	0.66 (0.43–1)	1.11 (0.23–5.40)	0.64
Artery media	5.25 (2.45–11.27)	6.87 (1.17–40.31)	0.99	1.11 (0.66–1.86)	1.62 (0.43–6.12)	0.73
Artery adventitia	13.08 (5.62–30.42)	9.06 (1.56–52.55)	0.38	1.22 (0.76–1.94)	2.46 (0.39–15.49)	0.94
Vein intima	4.99 (1.531–16.24)	4.31 (0.47–39.36)	0.69	0.67 (0.38–1.18)	1.66 (0.21–13.42)	0.39
Vein media	5.43 (2.12–13.91)	10.71 (2.14–53.47)	0.45	0.70 (0.47–1.05)	1.45 (0.21–9.96)	0.66
Vein adventitia	17.94 (6.84–47.06)	11.42 (2.04–64.15)	0.69	1.24 (0.73–2.12)	2.26 (0.31–16.33)	0.87

Ki67+ cells/mm^2

Data are presented as geometric means with 95% confidence intervals (95% CI). Statistical comparisons were performed using the Mann–Whitney U test. Statistical significance was defined as *P *< 0.05 (*) and *P *< 0.01 (**)

### α-smooth muscle actin (α-SMA) expression in vascular wall layers following ischemic insult

**Fig. 6:**
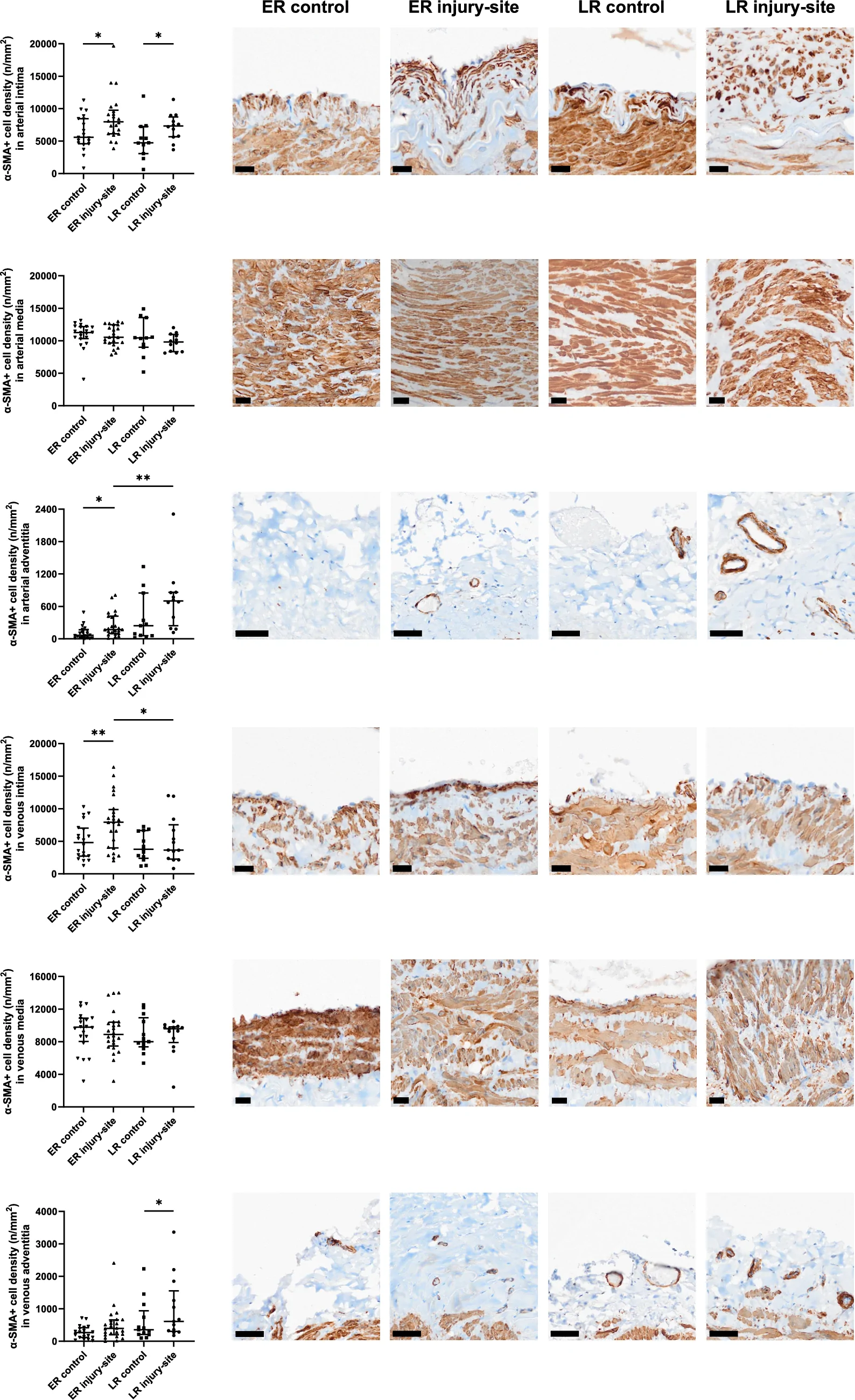
α-smooth muscle actin (α-SMA) expression in vascular wall layers of early (ER) and late reconstruction (LR) groups and their controls. α-SMA+ cells were identified with an α-SMA-antibody and quantified in each layer of the vessels. Representative images of all groups are provided adjacent to each graph. Scale bars: 50 μm for arterial and venous adventitia and 20 μm for arterial and venous media and intima. ^*^P < 0.05, ^**^P < 0.01 and ^***^P < 0.001. Middle line=median and whiskers=interquartile range (IQR). Statistical tests are reported in Tables 7 and 8. Images were taken with Olympus VS200 Slide Scanner Microscope

To address different cell populations in the blood vessels exposed to severe ischemia, we next explored cells expressing α-SMA. α-SMA is a marker for smooth muscle cells, pericytes and myofibroblasts in blood vessels [17–19]. α-SMA-expressing cells (α-SMA +) were present within all vessel wall layers in injury and control blood vessels of both study groups (Fig. 6). There are many α-SMA+ cells in the normal arterial intima and media, and one can identify a few α-SMA+ cells also in normal adventitia.

The α-SMA+ cell density was higher in intima and a bit lower in medial layer of the injury-site samples than in the controls within the study groups. However, more than twofold more α-SMA+ cells were identified in adventitia in early and late reconstruction groups injury-site samples than in the controls. In veins, more α-SMA–expressing cells were observed in the intimal layer of the early reconstruction group and in the adventitial layer of the late reconstruction group, than in their respective controls (Table 7).

**Table 7:** Comparison of α-SMA-positive cells between control and injury-site vessel samples in the early reconstruction (ER) and late reconstruction (LR) groups

	ER control	ER injury-site	*p*-value	LR control	LR injury-site	*p*-value
Artery intima	5587 (4592–8468)	7986 (6126–9765)	0.03*	4740 (3072–7251)	7335 (5667–8727)	0.04
Artery media	11,274 (10,349–12,172)	10,537 (9675–12,478)	0.47	10,443 (9014–13,574)	9807 (8328–10,969)	> 0.99
Artery adventitia	65 (18–168)	171 (99–418)	0.01*	243 (59–847)	701 (243–860)	0.23
Vein intima	4815 (2726–7027)	7924 (3985–9879)	< 0.01**	3793 (2380–6645)	3651 (2263–7553)	0.83
Vein media	9779 (8033–10,902)	8903 (7475–10,387)	0.89	8005 (7363–10,950)	9588 (7894–9823)	0.99
Vein adventitia	263 (118–419)	394 (200–657)	0.07	354 (207–938)	612 (303–1552)	0.03*

α-SMA+ cells/mm^2

Data are presented as median (IQR). Comparisons were performed using the Wilcoxon signed-rank test. Statistical significance was defined as *P *< 0.05 (*) and *P *< 0.01 (**)

Comparative analysis of early and late reconstruction groups showed more α-SMA+ cells in arterial adventitial layer of the injury-site samples in the late reconstruction group, but no statistically significant differences were observed in other arterial layers. The population of α-SMA expressing cells was more than two times greater in the intimal layer of the veins harvested from the early reconstruction group than in the late reconstruction group (p=0.04). No statistically significant differences were detected in α-SMA positive cell density in any layer of control samples between the study groups (Table 8).

**Table 8:** Comparison of α-SMA-positive cells in vessel walls between the early reconstruction (ER) and late reconstruction (LR) groups

	ER injury-site	LR injury-site	*p*-value	ER control	LR control	*p*-value
Artery intima	7986 (6126–9765)	7335 (5667–8727)	0.28	5587 (4592–8468)	4740 (3072–7251)	0.23
Artery media	10,537 (9675–12,478)	9807 (8328–10,969)	0.11	11,274 (10,349–12,172)	10,443 (9014–13,574)	0.40
Artery adventitia	171 (99–418)	701 (243–860)	< 0.01**	65 (18–168)	243 (59–847)	0.07
Vein intima	7924 (3985–9879)	3651 (2263–7553)	0.04*	4815 (2726–7027)	3793 (2380–6645)	0.46
Vein media	8903 (7475–10,387)	9588 (7894–9823)	> 0.99	9779 (8033–10,902)	8005 (7363–10,950)	0.37
Vein adventitia	394 (200–657)	612 (303–1552)	0.08	263 (118–419)	354 (207–938)	0.11

α-SMA+ cells/mm^2

Data are presented as median (IQR). Comparisons were performed using the Mann–Whitney U test. Statistical significance was defined as *P *< 0.05 (*) and *P *< 0.01 (**)

### Adventitial neo-vascularization in response to trauma-induced ischemia

**Fig. 7:**
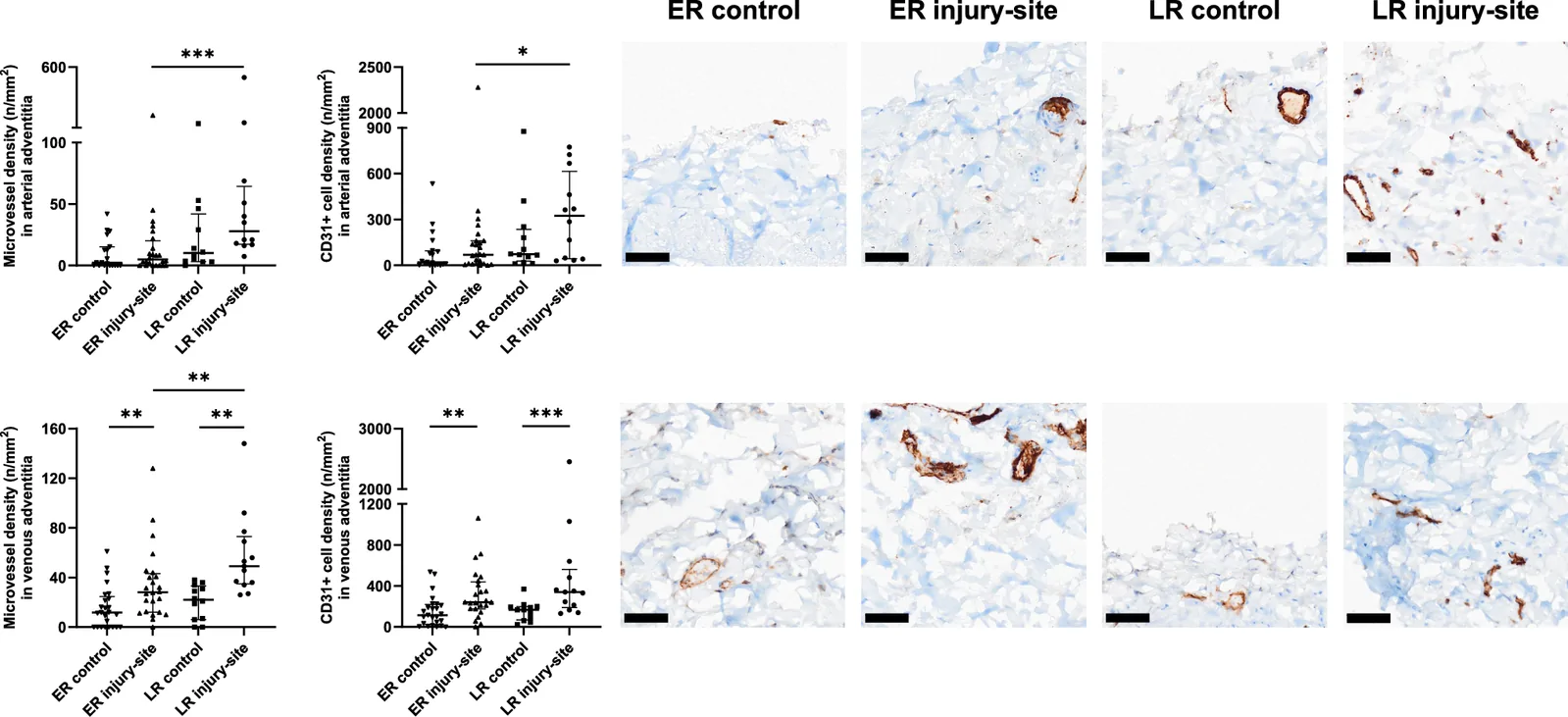
Adventitial angiogenesis of arterial and venous walls following trauma induced ischemic insult. Histological sections were stained with an endothelial antibody (CD31) and the number of endothelial cells and micro vessels were defined in the venous and arterial adventitia. Representative images of all groups are provided adjacent to each graph. Scale bars: 50 μm. ^*^P < 0.05, ^**^P < 0.01 and ^***^P < 0.001. Middle line=median and whiskers=interquartile range (IQR). Statistical tests are reported in Tables 9 and 10. Images were taken with Olympus VS200 Slide Scanner Microscope

Higher proliferative activity and population of α-SMA expressing cells in adventitial layer of injury-site samples along with microscopically suspected higher micro-vessel density were suggestive of angiogenesis, i.e. collateral blood vessel formation in response to ischemia. To address the angiogenesis, the biopsies were stained with endothelial cell marker cluster of differentiation 31 (CD31) to analyse adventitial angiogenesis (Fig. 7). We determined both the endothelial cells as well as the number of blood vessels in the adventitia. The number of blood vessels and endothelial cells (CD31 positive cells/blood vessels per adventitial square-millimetre) was 2-fold higher in the injury-site arteries than in controls in the early reconstruction group (NS). The number of blood vessels and endothelial cells was more than two times higher in the injury-site veins than in the controls in the early reconstruction group (*P* < 0.01). Blood vessels and endothelial cells were increased > twofold in adventitia of both arteries and veins of late reconstruction group, however, the difference was statistically significant in veins only (*p* ≤ 0.01, Table 9). Comparison of early and late reconstruction groups demonstrated that there were more adventitial blood vessels in late reconstruction group arteries and veins than in the early reconstruction group. There were no statistically significant differences in the number of blood vessels or endothelial cells between the control samples (Table 10).

**Table 9:** Comparison of microvessel density, assessed by CD31 immunostaining, between control and injury-site samples in the early reconstruction (ER) and late reconstruction (LR) groups

	ER control	ER injury-site	*p*-value	LR control	LR injury-site	*p*-value
Vessels in arterial adventitia^1^	2 (0–15)	5 (0–20)	0.45	10 (3–42)	28 (17–64)	0.10
CD31+ cells in arterial adventitia^2^	20 (5–95)	69 (10–161)	0.08	73 (28–234)	324 (43–616)	0.08
Vessels in venous adventitia^1^	12 (0–25)	28 (12–43)	< 0.01**	22 (6–33)	49 (35–73)	< 0.01**
CD31+ cells in venous adventitia^2^	115 (25–218)	242 (177–439)	< 0.01**	167 (69–198)	341 (189–562)	< 0.01**

^1^CD31 positive vessels/mm^2

^2^CD31+ cells/mm^2

Data are presented as median (IQR). Comparisons were performed using the Wilcoxon signed-rank test. Statistical significance was defined as *P *< 0.05 (*) and *P *< 0.01 (**)

**Table 10:** Comparison of microvessel density, assessed by CD31 immunostaining between the early reconstruction (ER) and late reconstruction (LR) groups

	ER injury-site	LR injury-site	*p*-value	ER control	LR control	*p*-value
Vessels in arterial adventitia^1^	5 (0–20)	28 (17–64)	< 0.01**	2 (0–15)	10 (3–42)	0.05
Endothelial cells in arterial adventitia^2^	69 (10–161)	324 (43–616)	0.02*	20 (5–95)	73 (28–234)	0.10
Vessels in venous adventitia^1^	28 (12–43)	49 (35–73)	< 0.01**	12 (0–25)	22 (6–33)	0.26
Endothelial cells in venous adventitia^2^	242 (177–439)	341 (189–562)	0.35	115 (25–218)	167 (69–198)	0.53

^1^CD31+ vessels/mm^2

^2^CD31+ cells/mm^2

Data are presented as median (IQR). Comparisons were performed using the Wilcoxon signed-rank test. Statistical significance was defined as *P *< 0.05 (*) and *P *< 0.01 (**)

**Fig. 8:**
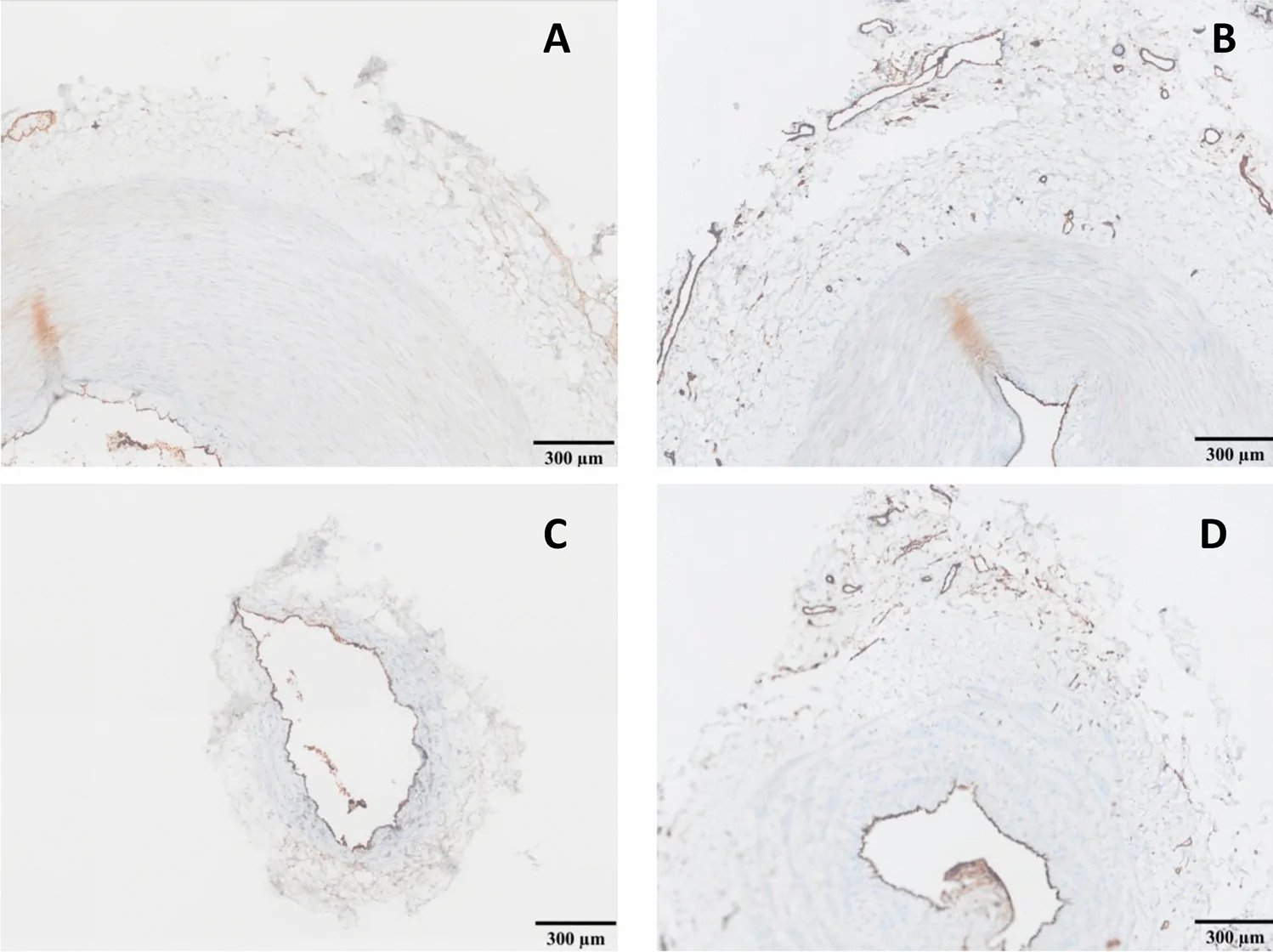
Arterial and venous walls harvested from the same patient at the same anatomical location, but at different time points, show progressive adventitial angiogenesis. Panels **A**–**B** represent arterial walls, and **C**–**D** venous walls, harvested on day 7 (**A**, **C**) and day 45 (**B**, **D**) following injury. Newly formed blood vessels detected with CD31-antibody are observed in the later samples

**Fig. 9:**
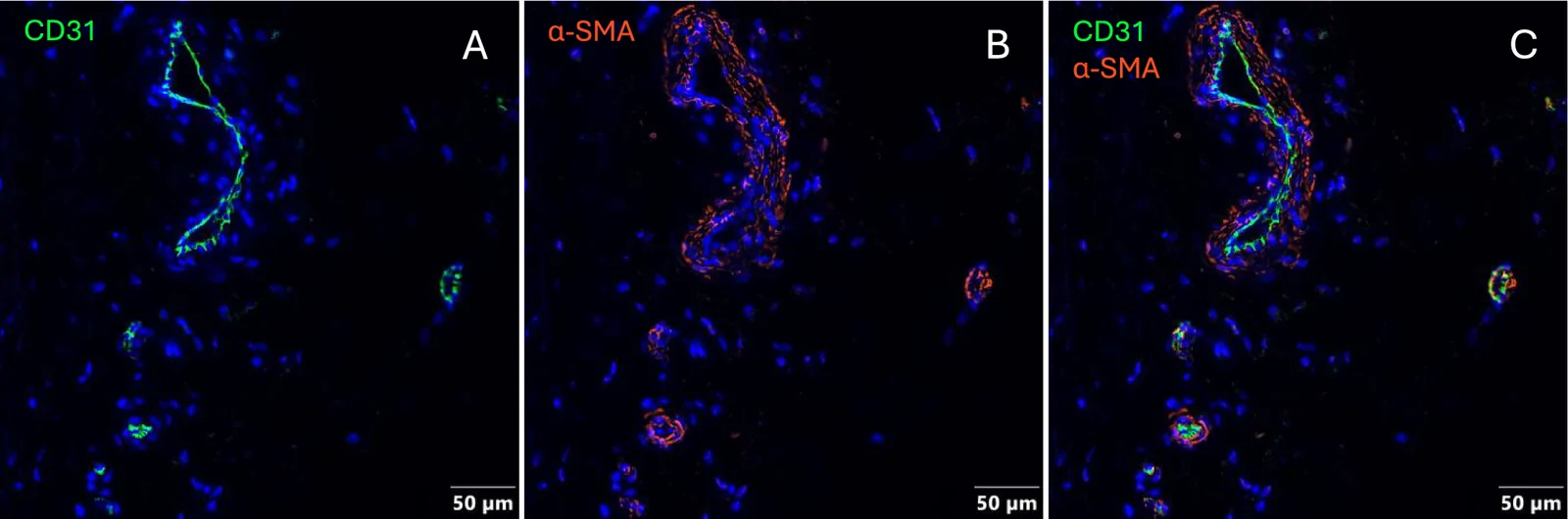
Immunofluorescence analysis of neovascularization in day 45 arterial samples. Immunofluorescence staining demonstrates the presence of newly formed, functional blood vessels within the adventitia. These vessels are characterized by open lumens lined with CD31-positive endothelial cells and surrounded by α-SMA–positive mural cells. **A** CD31 staining highlights endothelial cells. **B** α-SMA staining identifies medial smooth muscle cells. **C** Merged staining of CD31 and α-SMA confirms the structural organization of newly formed vessels

To best illustrate ongoing adventitial angiogenesis, we present CD31-stained anterior tibial artery and concomitant vein samples collected from the same patient at the identical anatomical location within different time points: first on day 7 post-injury during free flap reconstruction and then on day 45 post-injury during below-knee amputation, after failed limb salvage attempt. Notably, substantial adventitial blood vessel formation is evident in the latter specimens both in injury-site arteries (18 vs. 8 adventitial vessels/mm^2^; 375 vs. 112 CD31 positive cells/mm^2^) and veins (40 vs. 0 adventitial vessels/mm^2^; 199 vs. 0 CD31 positive cells/mm^2^) (Fig. 8). Furthermore, immunofluorescence staining of day-45 arterial samples with CD31 and α-SMA confirmed the presence of newly formed, functional blood vessels within the adventitia, characterized by open lumens lined with CD31 positive endothelial cells and surrounded by α-SMA+ medial smooth muscle cells (Fig. 9).

## Discussion

Oxygen is fundamentally important for most living organisms, and maintaining oxygen homeostasis represents a key physiological challenge in humans. Consequently, oxygen deprivation—hypoxia—is a critical factor in the pathophysiology of numerous human diseases. However, current knowledge of how the vascular wall responds to trauma and hypoxia in the surrounding tissues is largely derived from experimental animal models and post-mortem human studies, with limited data available from living human tissues. To address the paucity of data on the response to trauma and hypoxia, we have collected biopsies from arteries and veins from patients who had free-flap transfer performed on their lower extremity open fracture either at the early or late phase in a prospective, controlled study. Our study demonstrates that the early responses to injury and ischemia are neointima formation and the initiation of angiogenesis (collateral blood vessel formation) from the venous blood vessels, whereas the collateral blood vessel formation from the arterial side can be seen at the late phase.

One of the key elements of vascular repair following injury/surgical procedure is intimal hyperplasia. [9, 20–23] Relatively slow, but progressive intimal hyperplasia takes place after endovascular procedures, especially after thermal balloon injury and stenting. The intimal hyperplasia gradually leads to restenosis after these surgical procedures [9, 10, 22]. This can be explained by the nature of endovascular stenting procedures where foreign material is introduced to blood vessel wall. This most probably triggers prolonged vascular inflammation and a cellular response resulting in progressive intimal hyperplasia. Thus, restenosis is more common after endovascular procedures than at the anastomotic sites of autograft bypasses. [6, 7, 24–26] Contrary to the aforementioned findings on surgical situations and experimental injury models where the blood vessel itself is injured, the results of this study, after an external trauma induced ischemic insult, show that human vessel wall intimal hyperplasia has a sudden onset and reaches its peak within a week after the injury, but it does not progress further. [27] It is plausible that the rapid development of intimal hyperplasia observed in the early reconstruction group reflects mild medial injury, thereby facilitating extensive and immediate smooth muscle cell migration, proliferation, and extracellular matrix accumulation in contrast to endovascular interventions that often result in more severe medial damage initially, that can potentially delay smooth muscle cell migration into the intima [22]. However, unlike the progressive hyperplasia observed after stenting, our findings suggest that intimal hyperplasia remains limited and does not progress significantly following injury and ischemia when no foreign material is introduced to the blood vessel. [5, 9, 10, 28–31]

The ECM accumulation in the arterial intima was around 2-fold higher at injury site than in the control samples in the early and late reconstruction groups. Considering the data from the experimental intimal hyperplasia-models where the actual ECM production is the late event of the process our data suggests that the primary mechanism for the enhanced accumulation of ECM in the blood vessel could be exudation/leakage of plasma proteins from the circulation caused by the increased vascular permeability [32]. This likely occurs as the response to acute ischemia by hypoxia inducible factor-1α (HIF-1α) mediated induction of vascular endothelial growth factor (VEGF). [33] VEGF, in turn, is the growth factor responsible not only for angiogenesis, but also for the increased leakage by enhanced vascular permeability after the injury. [33–35]Along these lines, the intimal ECM accumulation took place only in arteries, but not in veins of the injury-site. We propose that the veins respond to ischemia by initiating adventitial angiogenesis, but ECM accumulation, driven (most probably) by the increased vascular permeability, does not seem to take place in them. These results highlight different acute responses to ischemia by veins and arteries.

Proliferative activity and a-SMA+ cell dynamics in early reconstruction group injury-site samples might reflect ongoing cellular migration into arterial and venous wall intima layers and proliferation resulting in acute intimal hyperplasia. [26, 29, 36–38] Lower numbers of α-SMA+ cells in the media alongside significantly higher numbers of α-SMA+ cells in the intima of early reconstruction group injury site samples relative to controls, suggests that medial vascular smooth muscle cells are likely responsible for intimal hyperplasia. This process is characterized by active migration from the media to the intima and subsequent proliferation in response to trauma, which is in line with findings from previous studies. [10, 26, 29, 30, 37, 38]

In contrast, in the late reconstruction group, proliferative activity within the intimal and medial layers of injury site arteries and veins was not statistically different from controls, contrasting with the early reconstruction group, where notably higher proliferative activity was observed across all vessel wall layers with strong statistical significance (p<0.01). Furthermore, the comparative analysis of intimal thickness between early and late reconstruction groups did not reveal statistically significant differences in either arterial or venous intimal thickness. Collectively, our findings suggest that structural changes contributing to intimal hyperplasia occur rapidly following injury and do not continue to progress during later stages.

Intensive vascular remodelling was also noticed in arterial and venous adventitia. Both the relatively high cell proliferation and the accumulation of α-SMA+ cells were identified in the injury-site samples. Interestingly, we identified more blood vessels in the adventitia of the veins in the early reconstruction group, but not in the arteries. However, both arteries and veins of the injury site showed more blood vessels in adventitia than their controls in the late reconstruction group. These results lend support to the concept that the venous endothelial cells are the primary source of endothelial cells responsible for angiogenic expansion in disease settings. [35] Remarkably, venous endothelial cells are the only type of endothelial subtype capable of responding to hypoxia-induced angiogenic signalling to form the new vascular bed. [35, 39–41]

This study has several notable strengths. First, we are not aware of any previous study where arterial and venous biopsies had been collected after major ischemic insult to human extremity. Furthermore, we collected control blood vessel samples of matching size from the site of free flap harvest essentially providing genetically identical control samples for each specimen. Our study design guarantees that the responses seen in the blood vessels are secondary to the ischemia induced in their surrounding tissues by major trauma. However, we acknowledge that although ischemia (driven by extensive soft tissue trauma surrounding the vessels) is most probably the main driver for vascular remodelling in our study, it is clear that multiple other factors such as inflammation, certain degree of infection in some cases, general tissue/wound healing response, altered hemodynamics and the previous surgical procedure (in the LR group) also contributed directly to the measured remodelling responses in the blood vessels. Second, the prospective study design ensures even patient selection and adherence to the study methodology applied throughout the entire patient’s series. Third, by comparing blood vessels exposed to ischemia with healthy control biopsies obtained from the matched blood vessels from each patient, we minimized interindividual variability and enhanced the validity of our findings. Fourth, the inclusion of specimens harvested at varying time points post-injury allowed for the characterization of dynamic changes within vessel wall, which indicates that a certain blood vessel remodelling pattern exists in human blood vessels in response to ischemia.

Nevertheless, several limitations should be acknowledged. Although we examined 40 patients (divided into groups of 26 and 14 patients) with severe open fractures, the relative rarity of this severe trauma may have influenced the findings. Additionally, heterogeneity among patients—including differences in age, sex, comorbidities and other individual factors—may have influenced the results. However, most of the patients were young, healthy males and to reduce potential bias and heterogeneity, we excluded all patients with comorbidities such as diabetes. Finally, variability in both the extent of the vascular injury and the severity of associated soft-tissue trauma may have introduced sampling bias. Although we collected the biopsies from the viable, pulsating part of the vessel, we cannot rule out the possibility that some vessel specimens were obtained from regions where they were directly affected by the blunt trauma to lower extremity (such as direct bumper contusion), thereby amplifying the apparent vascular response. The risk of this is considered low, because all samples were harvested from macroscopically healthy sites suitable for microvascular anastomosis.

We acknowledge that relevant clinical outcome measures, which could be compared to the histopathological changes found in this study, would increase the relevance of these findings. However, given the relatively small number of patients and the excellent clinical outcomes of this prospective cohort—with no major vascular complications and free flap loss occurring in this series—we could not assess whether histopathological parameters were independently associated with clinical outcomes after adjustment for potential confounders. Larger studies with more clinical events would be needed to clarify the prognostic significance of these vascular morphological changes.

Future studies in this field should investigate the cellular and molecular mechanisms underlying intimal hyperplasia, with a focus on identifying therapeutic targets or pharmacologic agents to prevent its development. Intimal hyperplasia is a leading cause of luminal restenosis following endovascular procedures. We demonstrate that it also occurs in lower extremity vessels after major blunt trauma, indicating its potential to contribute to vascular remodelling and, potentially, luminal narrowing. However, the intimal thickening observed in this study should be interpreted as morphological evidence of vascular remodelling rather than direct evidence of clinically significant ischemia or impaired tissue perfusion. Further studies incorporating functional vascular assessments are needed to determine the clinical significance of these structural changes. Moreover, the role of adventitial angiogenesis (collateral blood vessel formation) and its long-term impact on vessel wall patency in the context of traumatic injury remain insufficiently understood and warrant further investigation. In the absence of prospective studies in humans, this study adds fundamental knowledge on how trauma and ischemia-induced vascular response and remodeling of human vessel walls take place. Moreover, our study defines the sequelae of ongoing changes from the acute post-injury phase to long-term structural adaptations occurring over months to years following injury.

## Conclusions

Our findings demonstrate that major ischemic insult caused by blunt trauma to the lower extremity induces active remodelling across all layers of the vascular wall. The earliest and most prominent structural changes are intimal hyperplasia, followed by adventitial angiogenesis originating from the veins, highlighting a temporally coordinated vascular response to ischemic injury.

## Supplementary Information

Below is the link to the electronic supplementary material.

Supplementary file1

## Data Availability

No datasets were generated or analysed during the current study.
