# Supplementary material for: Intimal hyperplasia and adventitial angiogenesis originating from veins are vascular remodelling responses in large vessels after major lower extremity trauma induced ischemia
Source: Angiogenesis. 2026 Sep 3;29(4):68. doi: 10.1007/s10456-026-10089-x (PMC13541956; doi:10.1007/s10456-026-10089-x)
Supplement: Supplementary file 1 [file 10456_2026_10089_MOESM1_ESM.docx]

*Table S1. Supplemental Digital Content. Detailed characteristics of the study population.*

| **Case** | **Age** | **Group** | **Comorbidities, smoking status** | **Reconstruction and vessel harvest day (post-injury)** | **Indication for soft tissue reconstruction** | **Free flap** | **Zone of injury sample origin** | **Control sample origin** |
| --- | --- | --- | --- | --- | --- | --- | --- | --- |
| **1** | 62 | Early reconstruction | Smoker | 6 | Open tibial fracture IIIB | Latissimus dorsi | Posterior tibial | Thoracodorsal pedicle |
| **2** | 56 | Early reconstruction | - | 7 | Open tibial fracture IIIB | Latissimus dorsi | Anterior tibial | Thoracodorsal pedicle |
| **3** | 29 | Early reconstruction | - | 11 | Open tibial fracture IIIB | Gracilis | Posterior tibial | Gracilis pedicle |
| **4** | 48 | Early reconstruction | Smoker | 3 | Open tibial fracture IIIB | Gracilis | Posterior tibial | Gracilis pedicle |
| **5** | 62 | Early reconstruction | - | 6 | Open tibial fracture IIIB | Gracilis | Posterior tibial | Gracilis pedicle |
| **6** | 44 | Early reconstruction | Asthma | 4 | Open tibial fracture IIIB | Latissimus dorsi | Posterior tibial | Thoracodorsal pedicle |
| **7** | 33 | Early reconstruction | - | 1 | Open calcaneal fracture | ALT | Posterior tibial | Descending branch of lateral circumflex femoral vessels |
| **8** | 66 | Early reconstruction | - | 8 | Open tibial fracture IIIB | Latissimus dorsi | Posterior tibial | Thoracodorsal pedicle |
| **9** | 75 | Early reconstruction | Coronary artery disease | 3 | Open tibial fracture IIIB | Gracilis | Posterior tibial | Gracilis pedicle |
| **10** | 78 | Early reconstruction | Persistent atrial fibrillation | 7 | Open tibial fracture IIIB | Latissimus dorsi | Posterior tibial | Thoracodorsal pedicle |
| **11** | 29 | Early reconstruction | Smoker | 10 | Open tibial fracture IIIC | Latissimus dorsi | Medial superior genicular | Thoracodorsal pedicle |
| **12** | 23 | Early reconstruction | - | 10 | Open Lisfranc fracture | Latissimus dorsi | Dorsalis pedis | Thoracodorsal pedicle |
| **13** | 48 | Early reconstruction | - | 8 | Open tibial fracture IIIB | Gracilis | Posterior tibial | Gracilis pedicle |
| **14** | 31 | Early reconstruction | Smoker | 10 | Open tibial fracture IIIB | Gracilis | Posterior tibial | Gracilis pedicle |
| **15** | 28 | Early reconstruction | - | 3 | Open tibial fracture IIIB | Latissimus dorsi | Medial sural | Thoracodorsal pedicle |
| **16** | 19 | Early reconstruction | Smoker | 1 | Open tibial fracture IIIB | Latissimus dorsi | Posterior tibial | Thoracodorsal pedicle |
| **17** | 56 | Early reconstruction | Arterial hypertension | 3 | Open tibial fracture IIIB | Gracilis | Posterior tibial | Gracilis pedicle |
| **18** | 49 | Early reconstruction | sleep apnea | 7 | Open femur fracture IIIB | Latissimus dorsi | Branch of profunda femoris | Thoracodorsal pedicle |
| **19** | 51 | Early reconstruction | - | 13 | Open tibial fracture IIIB | Latissimus dorsi | Posterior tibial | Thoracodorsal pedicle |
| **20** | 31 | Early reconstruction | - | 9 | Open ankle fracture IIIB | ALT | Posterior tibial | Descending branch of lateral circumflex femoral vessels |
| **21** | 26 | Early reconstruction | Bicuspid aortic valve | 5 | Open tibial fracture IIIB | Gracilis | Posterior tibial | Gracilis pedicle |
| **22** | 38 | Early reconstruction | - | 7 | Foot crush injury | Latissimus dorsi | Anterior tibial | Thoracodorsal pedicle |
| **23** | 29 | Early reconstruction | - | 5 | Open tibial fracture IIIB | ALT | Posterior tibial | Descending branch of lateral circumflex femoral vessels |
| **24** | 50 | Early reconstruction | - | 4 | Open tibial fracture IIIB | ALT | Posterior tibial | Descending branch of lateral circumflex femoral vessels |
| **25** | 58 | Early reconstruction | - | 5 | Open tibial fracture IIIB | Latissimus dorsi | Posterior tibial | Thoracodorsal pedicle |
| **26** | 34 | Early reconstruction | heavy drug user | 6 | Open femur fracture IIIB | Latissimus dorsi | Popliteal | Thoracodorsal pedicle |
| **27** | 60 | Late reconstruction | - | 60 | Late infection following open ankle fracture | ALT | Anterior tibial | Descending branch of lateral circumflex femoral vessels |
| **28** | 56 | Late reconstruction | - | 80 | Late infection following open tibial fracture | LD | Posterior tibial | Thoracodorsal pedicle |
| **29** | 28 | Late reconstruction | - | 431 | Late infection following open tibial fracture | Gracilis | Posterior tibial | Gracilis pedicle |
| **30** | 58 | Late reconstruction | - | ~3650 | Chronic osteomyelitis (open tibial fracture in 2011) | LD | Posterior tibial | Thoracodorsal pedicle |
| **31** | 34 | Late reconstruction | - | 6570 | Chronic none healing wound after open tibial fracture | ALT | Anterior tibial | Descending branch of lateral circumflex femoral vessels |
| **32** | 24 | Late reconstruction | Smoker | 34 | Massive soft tissue injury of the lower extremity | ALT | Posterior tibial | Descending branch of lateral circumflex femoral vessels |
| **33** | 62 | Late reconstruction | - | 32 | Open tibial and calcaneal fracture, intramedullary abscess, infection | Gracilis | Posterior tibial | Gracilis pedicle |
| **34** | 57 | Late reconstruction | - | 28 | Infection after fracture fixation and ALT flap reconstruction | ALT | Anterior tibial | Descending branch of lateral circumflex femoral vessels |
| **35** | 49 | Late reconstruction | - | 17 | Hardware exposure, infection following proximal tibia fracture | LD | Posterior tibial | Thoracodorsal pedicle |
| **36** | 21 | Late reconstruction | - | 19 | Traumatic amputation of the lower extremity | LD | Popliteal | Thoracodorsal pedicle |
| **37** | 30 | Late reconstruction | Asthma, endometriosis, smoker | 72 | Progressive soft tissue necrosis following open tibial and fibular fracture | LD | Posterior tibial | Thoracodorsal pedicle |
| **38** | 38 | Late reconstruction | Smoker | 45 | Infection after fracture fixation | Amputation | Anterior tibial | Thoracodorsal pedicle (control samples harvested at the time of LD flap reconstruction). |
| **39** | 51 | Late reconstruction | Palmoplantar pustulosis, smoker | 48 | Infection after lower extremity fracture fixation | Gracilis | Posterior tibial | Gracilis pedicle |
| **40** | 63 | Late reconstruction | - | 380 | Osteomyelitis after open fracture repair | ALT | Posterior tibial | Descending branch of lateral circumflex femoral vessels |
